# Incidence of Human Herpesvirus 8 (HHV-8) infection among HIV-uninfected individuals at high risk for sexually transmitted infections

**DOI:** 10.1186/1471-2334-7-143

**Published:** 2007-12-05

**Authors:** Massimo Giuliani, Paola Cordiali-Fei, Concetta Castilletti, Aldo Di Carlo, Guido Palamara, Stefano Boros, Giovanni Rezza

**Affiliations:** 1 Struttura Complessa di Dermatologia Infettiva, Istituto Dermatologico San Gallicano (IRCCS), Rome, Italy; 2 Laboratorio di Patologia Clinica e Microbiologia, Istituto Dermatologico San Gallicano (IRCCS), Rome, Italy; 3 Laboratorio di Immunologia Cellulare, Laboratorio di Virologia, Istituto Nazionale di Malattie Infettive, L. Spallanzani (IRCCS), Rome, Italy; 4 Reparto di Epidemiologia, Dipartimento di Malattie Infettive, Parassitarie e Immunomediate (MIPI), Istituto Superiore di Sanità, Rome, Italy

## Abstract

**Background:**

The occurrence of, and risk factors for, HHV-8 infection have yet to be definitively determined, particularly among heterosexual individuals with at-risk behavior for sexually transmitted infections (STI). The objective of this study was to estimate the incidence and determinants of HHV-8 infection among HIV-uninfected individuals repeatedly attending an urban STI clinic.

**Methods:**

Sera from consecutive HIV-uninfected individuals repeatedly tested for HIV-1 antibodies were additionally tested for HHV-8 antibodies using an immunofluorescence assay. To identify determinants of HHV-8 infection, a nested case-control study and multivariate logistic regression analysis were performed.

**Results:**

Sera from 456 HIV-uninfected individuals (224 multiple-partner heterosexuals and 232 men who have sex with men (MSM]) were identified for inclusion in the study. The HHV-8 seroprevalence at enrollment was 9.4% (21/224; 95% C.I.: 6.0–14.2%) among heterosexuals with multiple partners and 22.0% (51/232; 95% C.I.: 16.9–28.0%) among MSM. Among the 203 multiple-partner heterosexuals and 181 MSM who were initially HHV-8-negative, 17 (IR = 3.0/100 p-y, 95% C.I.: 1.9 – 4.8) and 21 (IR = 3.3/100 p-y, 95% C.I:.2.1 – 5.1) seroconversions occurred, respectively. HHV-8 seroconversion tended to be associated with a high number of sexual partners during the follow-up among MSM (> 10 partners: AOR = 3.32 95% CI:0.89–12.46) and among the multiple-partner heterosexuals (> 10 partner; AOR = 3.46, 95% CI:0.42–28.2). Moreover, among MSM, HHV-8 seroconversion tended to be associated with STI (AOR = 1.80 95%CI: 0.52–7.96).

During the study period the HIV-1 incidence was lower than that of HHV-8 among both groups (0.89/100 p-y among MSM and 0.95/100 p-y among multiple-partner heterosexuals).

**Conclusion:**

The large difference between the incidence of HHV-8 and the incidence of HIV-1 and other STIs may suggest that the circulation of HHV-8 is sustained by practices other than classical at-risk sexual behavior.

## Background

The modes of transmission of human herpesvirus 8 (HHV-8), also known as "Kaposi's Sarcoma-associated Herpesvirus" (KSHV), have yet to be clearly established. Several studies have suggested that it may be transmitted sexually, although the role of specific sexual practices have yet to be conclusively defined. In areas of low endemicity, such as North America and Northern Europe, HHV-8 infection appears to be concentrated among men who have sex with men (MSM) at high risk of HIV-1 infection and other sexually transmitted infections (STI), whereas it is uncommon among persons who have not reached the age of sexual activity [1,2]. Moreover, several prevalence studies among MSM have reported an association between HHV-8 infection and receptive anal sex, the number of sexual partners, HSV-2 infection, hepatitis B virus infection, a history of syphilis, and HIV-1 infection, suggesting that the modes of transmission of HHV-8, among MSM, are similar to those for common STIs [3-7].

However, the results of recent incidence studies among MSM suggest that oro-genital sex, rather than receptive anal sex, is an important mode of HHV-8 transmission [8-10]. That the infection may be transmitted through saliva or other types of casual contact has been suggested by seroepidemiological studies which have reported a high prevalence of HHV-8 infection among children in Sub-Saharan Africa and, to some extent, in Mediterranean countries [11,12]. The hypothesis of transmission through saliva is also supported by the results of studies showing that HHV-8 DNA sequences are more likely to be detected in saliva or in mouth swabs than in semen or cervical-vaginal swabs [13-16].

With regard to the potential for sexual transmission among heterosexual individuals, little information is available. Among heterosexual men living in areas where the infection is endemic, a recent study has suggested that sexual factors may play an important role [17]. Moreover, cross-sectional studies of women with (or at risk of) HIV-1 infection have identified a history of syphilis, HIV-1 infection, older age, black race, intravenous drug use, gonorrhea, and alcohol abuse as correlates of HHV-8 infection, whereas other studies have shown no evidence of transmission through sexual contact [7,18-21].

Regarding the incidence of HHV-8 infection, two large studies conducted among MSM, one in the U.S.A. and the other in the Netherlands, have reported similar rates: 3.6 and 3.8/100 person-years, respectively, whereas there are no published estimates of the incidence among non-intravenous-drug-using heterosexual men or women [5,22].

To estimate the prevalence and incidence of HHV-8 infection and to identify risk factors for seroconversion and correlates of infection, we conducted a retrospective longitudinal study using frozen serum samples from non-drug-using MSM and heterosexual men and women with multiple partners who had repeatedly undergone HIV-1 testing at an important screening site in Rome, Italy.

## Methods

### Study population

The study participants were non-drug-using MSM and multiple-partner heterosexuals who, between July 1, 1992 and June 30, 1999, had at least twice undergone voluntary counseling & testing for antibodies against HIV-1 at an inner-city STI Clinic. The study site is the largest STI clinic in Rome, Italy, with a documented expertise in STI screening and control programs target to at-risk populations. Multiple-partner heterosexuals were defined as individuals who reported that they had had at least 3 sexual partners of the opposite sex in the 12 months prior to the first test in the study period and that they had never injected recreational drugs or engaged in homosexual intercourse in their lifetime.

Information on behavioral risk factors for the transmission of HIV-1 was taken from the clinic's computerized database. Data were collected by means of a standard interview conducted during the pre-HIV-test counseling session.

Given the evidence of a measurable risk of HHV-8 transmission through blood or blood derivatives, we excluded all individuals with a history of blood transfusion or the use of blood-derivatives before the first HIV-test or during the follow-up period [23-25].

### Study design

A retrospective longitudinal study was conducted using coded serum samples that had been collected from all eligible individuals and stored at -80°C. The study consisted of a cross-sectional phase for assessing the prevalence of HHV-8 infection at enrollment and, among participants who tested HHV-8-negative at enrollment, a longitudinal phase for assessing the incidence of HHV-8 infection.

### Laboratory procedures

#### Anti-HHV-8 immunofluorescence assay

Sera from all participants were tested for antibodies to lytic antigens of HHV-8 using an immunofluorescence assay (IFA) based on BCBL-1 cell line. In accordance with Andreoni et al., sera reactive at a 1:20 dilution were considered as positive, so as to attain the optimal levels of sensitivity and specificity of the test, as demonstrated in a previous multicenter study [26,27].

For each antibody assay, aliquots of pooled negative serum samples from 10 children (age 0–6 years) and of positive serum samples from 5 HIV-negative KS patients were included as additional controls.

#### HIV-1 antibody assay

Sera were tested for HIV-1 antibodies using an enzyme-linked immunoabsorbent assay (ELISA Genelavia Mix, Pasteur, France). Reactive sera were confirmed using a Western blot test (Western Blot, NevLav Blot I, Sanofi-Pasteur, France).

### Data analysis

The prevalence of HHV-8 infection was calculated based on serostatus at enrollment, and the correlates of prevalent HHV-8 infection were assessed using 2 × 2 table analysis. The incidence rates were calculated using the person-time method and were expressed in per 100/person year (p-y) of follow-up. The follow-up period was considered as the interval of time between the date of the first serum sample and the date of the last sample. For HHV-8 seroconverters, the follow-up period was defined as the time elapsed from the first HHV-8-negative sample to the midpoint in time between the last HHV-8-negative sample and the first HHV-8 positive sample.

To identify risk factors for HHV-8 infection, seroconverters were compared with persistently HHV-8 negative individuals for selected clinical and behavioral characteristics, using a nested case-control analysis. A multivariate logistic regression analysis was applied to assess the independent role of the variables. All of the demographic, behavioral and clinical data for the study participants were extracted from the HIV-1 screening computerized database.

The number of years of sexual activity was considered as a dichotomous variable, determined using the median value as the cut-off (less than the median value vs. greater than or equal to this value).

## Results

### HHV-8 prevalence

During the study period, 456 HIV-uninfected Caucasian individuals who consecutively visited the STI clinic were eligible for enrollment in the study: 224 were multiple-partner heterosexuals (185 males and 39 females) and 232 were MSM.

Overall, 72 of the 456 study participants (15.8%; 95% CI: 12.6%–19.5%) were HHV-8-seropositive upon enrollment. The median age was 36 years (range: 20–70) for prevalent HHV-8-infected individuals and 32 years (range: 17–69) for HHV-8 uninfected individuals. The seroprevalence of HHV-8 infection at enrollment was 9.4% (21/224; 95% CI: 6.0%–14.2%) among HIV-uninfected multiple-partner heterosexuals and 22.0% (51/232; 95% CI: 16.9–28.0%) among HIV-uninfected MSM (Table 1).

**Table 1:** Behavioral and virological characteristics of multiple-partner heterosexuals and of men who have sex with men (MSM) by HHV-8 antibody status at the enrollment.

	**MULTIPLE-PARTNER HETEROSEXUALS**		**MSM**	
	
	**HHV-8+/T**	**COR (95% C.I.)**	**HHV-8+/T**	**COR (95% C.I.)**
**All**	21/224 (9.4%)	(6.0%–14.2%)	51/232 (22.0%)	(16.9%–28.0%)
**Gender**				
Females	3/39 (7.7)	1.0	-	-
Males	18/185 (9.7)	1.29 (0.35–7.21)	51/232 (22.0)	-
**Age group (years)**				
< 25	6/42 (14.3)	1.0	3/25 (12.0)	1
25–35	7/96 (7.3)	0.47 (0.13–1.84)	20/99 (20.2)	1.86 (0.48–10.60)
> 35	8/86 (9.3)	0.62 (0.17–2.33)	28/108 (25.9)	2.57 (0.69–14.32)
**Age at sexual debut **§				
≥ 17 years	7/102 (6.9)	1.0	22/116 (19.0)	1
< 17 years	9/87 (10.3)	1.57 (0.50–4.97)	23/84 (27.4)	1.61 (0.78–3.33)
**Yrs. of sexual activity**§				
Median (range)		14 (1–54)	16 (2–45)	
< median value	10/99(10.1)	1.0	19/101 (18.8)	1
≥ median value	6/90(6.7)	0.64 (0.18–2.04)	26/99 (26.3)	1.54 (0.75–3.17)
No. of partners*				
< 3	0/0 (-)	-	7/41 (17.1)	1
3	8/62 (12.9)	1.0	6/30(20.0)	1.27 (0.31–4.72)
4–5	1/37 (2.7)	0.19 (0.00–1.52)	16/48 (33.3)	2.43 (0.80–7.57)
> 5	9/41 (21.9)	1.90 (0.59–6.11)	5/13 (38.5)	3.04(0.58–14.61)
History of STI**				
Negative	12/143 (8.4)	1.0	22/109(20.2)	1
Positive	9/81 (11.1)	1.36 (0.50–3.68)	29/123(23.6)	1.22 (0.62–2.30)
**History of cervical gonorrhea**				
Negative	3/38 (7.9)	1.0	-	-
Positive	0/1 (0.0)	N.A.	-	-
**History of gonorrhea**				
Negative	13/156 (8.3)	1.0	37/184(20.1)	1
Positive	5/29 (17.2)	2.29 (0.64–7.87)	14/48 (29.2)	1.64 (0.74–3.57)
**History of syphilis**				
Negative	15/194 (7.7)	1.0	37/156 (23.7)	1
Positive	6/30 (20.0)	2.98 (0.92–9.37)°	14/76 (18.4)	0.73 (0.34–1.53)

**° **p < 0.05.

COR: Crude Odds Ratio; N.A.: not applicable; *During the previous year; missing data for 84 Multiple partner heterosexual and 100 MSM; **The STIs investigated were: genital warts, unspecific urethral/vaginal infections, gonorrhea, syphilis and genital herpes. §Missing data for 32 MSM and 35 Multiple-partner heterosexuals.

The persistence of positivity for HHV-8 antibodies over time, was assessed among 53 out of the 72 individuals who were HHV-8 positive at recruitment. Using serum samples collected for sequential HIV-antibody test, HHV-8 antibodies positivity was confirmed for 52 out of 53 HHV-8 positive individuals (98.1%).

Among the multiple-partner heterosexuals, the seroprevalence was higher among males (9.7%) than among females (7.7%). No significant statistical associations were found between HHV-8 seropositivity and the investigated behavioral variables. Nonetheless, higher HHV-8 rates were observed among younger multiple-partner heterosexuals (14.3% for age < 25 years) compared to those who were older (Table 1). Moreover, HHV-8 seroprevalence tended to be higher for persons with: age at first sexual intercourse of less than 17 years (OR = 1.57; 95% CI: 0.50–4.97); more than five sexual partners in the previous year (OR = 1.90; 95% CI: 0.59–6.11); syphilis (OR = 2.98, 95% CI: 0.92–9.37; p < 0.05); and, among males, a history of urethral gonorrhea (OR = 2.29, 95% CI: 0.64–7.87) (Table 1).

Among MSM, HHV-8 seroprevalence tended to increase with age (OR = 1.86, 95% CI: 0.48–10.60 for the 25–35-year age group, and OR = 2.57, 95% CI: 0.69–14.32 for the > 35-year age group); it also increased with the number of years of sexual activity (OR = 1.54, 95% CI: 0.75–3.17 for more than 16 years) and with younger age at first sexual contact (less than 17 years) (OR = 1.61, 95%CI:0.78–3.33); however, none of these associations was statistically significant (Table 1). Moreover, among MSM, HHV-8 infection also tended to be associated with a higher number of sexual partners in the previous year and with history of STIs, particularly gonorrhea (Table 1).

### HHV-8 incidence

Overall, 384 of the study participants (203 multiple-partner heterosexuals and 181 MSM) were initially negative for HHV-8 antibodies and were thus included in the longitudinal seroincidence study. Of these participants, 348 (90.6%) were males. The median age at enrollment was 31 years (range: 17–69) for multiple-partner heterosexuals and 33.5 years (range: 19–61) for MSM. The median duration of follow-up was 2.8 years overall (range: 0.4–7.0; it was 2.6 years (range: 0.4–7.0) for multiple-partner heterosexuals and 3.2 years (range: 0.5–6.7) for MSM.

Overall, 38 individuals seroconverted during the follow-up period, of whom 36 (94.7%) were men. The HHV-8 incidence was 3.2/100 person-years (p-y) overall; it was 3.0/100 p-y (95% C.I.: 1.9–4.8) for multiple-partner heterosexuals and 3.3/100 p-y for MSM (95% C.I.:2.1–5.1). (Table 2).

**Table 2:** Seroincidence of HHV-8 infection among 384 individuals repeatedly tested for HIV-1 by exposure group and gender.

	**Total cases**	**Person-years (p-y)**	**Number of seroconversions**	**Incidence rate x100/p-y**	95% C.I.*
**ALL**	**384**	**1,193**	**38**	**3.2**	**2.3 – 4.4**
**M. P. H.**	203	564	17	3.0	1.9 – 4.8
*Males*	*167*	*476*	*15*	*3.1*	*1.9 – 5.1*
*Females*	*36*	*88*	*2*	*2.3*	*0.5 – 9.2*
**MSM**	181	629	21	3.3	2.1 – 5.1

MSM: men who have sex with men; MPH: multiple-partner heterosexuals. * C.I.: confidence intervals.

During the study period, 11 of the 384 individuals who were negative for HIV-1 and HHV-8 and included in the longitudinal study seroconverted for HIV-1, for an overall seroincidence of 0.92/100 p-y. The incidence of HIV-1 was lower than that of HHV-8 among both multiple-partner heterosexuals (0.89/100 p-y vs. 3.1/100 p-y, respectively) and MSM (0.95/100 p-y vs. 3.2/100 p-y, respectively). Moreover, for both groups, the incidence of each of the STIs investigated during the follow-up period (i.e., genital warts, unspecific urethritis, unspecific vaginal infections, gonococcal infections, syphilis, and genital herpes) was lower than the incidence of HHV-8 (data not shown).

Among multiple-partner heterosexuals, when comparing HHV-8 seroconverters to those who were persistently negative, no statistically significant differences were found in terms of gender, age, HIV-1 seroconversion, or occurrence of STIs (Table 3). Nonetheless, among multiple-partner heterosexuals, HHV-8 seroconversion was linearly associated with the number of sexual partners during follow-up (χ^2 ^for trend = 3.37; p = 0.07). This association, though not significant, was also shown in the logistic model (AOR = 3.46; 95% CI: 0.42–28.2 partners > 10).

**Table 3:** HHV-8 attack rate and OR (crude and adjusted) of HHV-8 seroconversion by behavioral and virological characteristics of 203 multiple-partner heterosexuals.

	**HHV-8 Serocon/T (%)N = 17/203**	**COR (95%C.I.)**	**AOR (95%C.I.)**	***P***
**Gender**				
Females	2/36 (5.5)	1.0	1.0	
Males	15/167 (9.0)	1.68 (0.36–15.77)	1.46 (0.31–6.87)	*.630*
Age **(years)				
< 25	3/25 (12.0)	1.0	1.0	
25–35	6/87 (6.9)	0.54 (0.13–2.35)	0.52 (0.11–2.37)	*.402*
> 35	8/91 (8.8)	0.71 (0.17–2.90)	0.56 (0.13–2.50)	*.451*
**STI during follow-up**				
None	13/165 (7.9)	1.0	1.0	
Any STI	4/38 (10.5)	1.38 (0.31–4.82)	1.56 (0.38–5.96)	*.586*
*Other STI *^	*3/31 (9.4)*	*1.25 (0.22–4.98)*	1.36 (0.35–5.32)	*.657*
*Syphilis*	*0/6 (-)*	*N.A*.	-	*-*
*Gonorrhea*	*1/1 (100.0)*	*Undetermined*	-	*-*
**HIV-1 during follow-up**				
Non-seroconverted	17/198 (8.6)	1.0	-	*-*
Seroconverted	0/5 (0.0)	N.A.	-	*-*
**No. sexual partners during follow-up**				
< 3	2/55 (3.6)	1.0	1.0	
3–5	6/74 (8.1)	2.34 (0.45–12.05)	2.45 (0.47–12.7)	*.288*
6–10	6/55 (10.9)	3.24 (0.62–16.84)	3.30 (0.62–17.4)	*.160*
> 10	3/19 (15.8)	4.96 (0.76–32.35)	3.46 (0.42–28.2)	*.247*

STI: Sexually Transmitted Infections; N.A.: not applicable;

** age at last test; ^ *Other STI *included: genital warts, unspecific urethral/vaginal infections, and genital herpes.

HHV-8 seroconversion was linearly associated with the number of sexual partners during follow-up also among MSM (χ^2 ^for trend = 3.38; p = 0.066), and the association, though not significant, was also shown in the logistic model (AOR = 3.32 95% CI: 0.89–12.46). Among MSM (Table 4), the risk of HHV-8 seroconversion also tended to be independently associated with gonococcal infection (AOR = 3.20 95% CI: 0.67–15.36).

**Table 4:** HHV-8 attack rates and OR (crude and adjusted) of seroconversion by behavioral and virological characteristics of 181 men who have sex with men.

	**HHV-8 Serocon/T (%)N = 21/181**	**COR (95%C.I.)**	**AOR (95% C.I.)**	***P***
**Gender**				
Females	-			
Males	21/181 (11.2)	*N.A*.	*N.A*.	
Age **(years)				
< 25	1/7 (14.3)	1.0	1.0	
25–35	9/71 (12.7)	0.87 (0.10–8.10)	0.69 (0.06–7.13)	*.758*
> 35	11/103 (10.7)	0.72 (0.10–7.52)	0.53 (0.05–5.70)	*.599*
**STI during follow-up**				
None	16/154(10.4)	1.0	1.0	
Any	5/27 (18.5)	1.96 (0.51–6.35)	1.80 (0.52–7.96)	*. 480*
*Other STI *^	*1/10 (10.0)*	*0.96 (0.02–7.76)*	1.13 (0.13–9.96)	*.913*
*Syphilis*	*1/7 (14.3)*	*1.44 (0.03–13.05)*	1.47 (0.15–14.13)	*.739*
*Gonorrhea*	*3/10 (30.0)*	*3.70 (0.56–18.11)*	3.20 (0.67–15.36)	*.146*
**HIV-1 during follow-up**				
Non-seroconverters	20/175(11.4)	1.0	1.0	
Seroconverters	1/6 (16.7)	1.55(0.03–14.87)	1.56 (0.16–15.69)	*.704*
**No. sexual partners during follow-up**				
< 3	4/55(7.3)	1.0	1.0	
3–5	4/43(9.3)	1.30 (0.31–5.56)	1.41 (0.32–6.13)	*.647*
6–10	4/36 (11.1)	1.59 (0.37–6.83)	1.50 (0.32–6.82)	*.601*
> 10	9/47 (19.1)	3.02 (0.86–10.54)	3.32 (0.89–12.46)	*.075*

STI: Sexually Transmitted Infections; N.A.: not applicable;

** age at the last test; ^ *Other STI *included: genital warts, unspecific urethral/vaginal infections and genital herpes.

## Discussion

Our study provides information on the incidence and prevalence of HHV-8 infection among HIV-uninfected individuals at high-risk of acquiring STIs. To the best of our knowledge, this is the first longitudinal study in an economically developed country to provide estimates of the incidence of HHV-8 infection among HIV-uninfected, yet promiscuous, non-drug-using heterosexual individuals.

HHV-8 seroprevalence at enrollment was higher among MSM, compared to multiple-partner heterosexuals, confirming the previously reported wider diffusion of the infection among homosexual men. However, it remains to be determined whether this difference was related to differences in the level of promiscuity or in specific sexual practices.

The HHV-8 seroprevalence among multiple-partner heterosexuals was lower than that reported in a study conducted among HIV-negative heterosexuals attending an STI clinic in another Mediterranean country (i.e., Spain), yet it was twice as high as that reported among HIV-negative non-African heterosexuals (4.6%) attending an STI clinic in London [28,7]. The seroprevalence among MSM was higher than the rates reported in other industrialized countries yet similar to those reported by other studies conducted among MSM in Italy [4,8,9,29-31].

With regard to the incidence of HHV-8 infection, the rate among MSM is consistent with the seroconversion rates reported by other longitudinal studies conducted among the same group. The incidence rate found in our study was similar to that among HIV-1-negative MSM in Seattle, USA (3.8/100 p-y) and somewhat higher than that among HIV-1-negative MSM in Amsterdam (2.6/100 p-y) [5,22]. As in other studies, none of the variables considered in our study as proxies of at-risk sexual behavior were found to have been associated with HHV-8 seroconversion among MSM [7,8]. To this regard, it should be mentioned that the study design was based on the investigation of a cohort of individuals evaluated to identify risk factors for HIV-1, and this may have limited the ability to detect determinants for HHV-8 infection.

Surprisingly, the HHV-8 seroincidence among heterosexual males was similar to that among MSM (3.1/100 vs. 3.3/100 p-y, respectively). Among heterosexuals, the seroincidence rate was lower among females than males (2.3/100 vs. 3.1/100 p-y), but the small number of events among females does not permit any robust comparison by gender.

There are no clear explanations for the inconsistency between incidence and prevalence data. In fact, the prevalence data, which are the expression of cumulative infections, seem to suggest that sexual practices related to male-to-male sex play an important role in the transmission of HHV-8. However, our seroincidence data did not reveal any excess risk related to male-to-male sex with respect to male-to-female sex. This could be due to recent behavioral changes or to a recent increase in the circulation of HHV-8 among multiple-partner heterosexuals, as indicated by the higher seroprevalence of HHV-8 among younger heterosexuals.

Furthermore, the higher risk of HHV-8 infection among both MSM and multiple-partner heterosexual males, compared with females, seem to suggests that insertive-penile intercourse may play an important role in the acquisition of the infection. This is somewhat consistent with studies that have identified penile-oral sex, a common sexual practice among both homosexual and heterosexual males, as a risk factor for HHV-8 infection and have suggested that the insertive partner is at greatest risk through contact with saliva [19,20]. In fact, HHV-8-DNA has frequently been detected in saliva, yet differently from HIV-1, it has rarely been found in semen or vaginal fluids [13,15,16,32].

Since it is well known that gonococcal uretrithis is efficiently acquired through insertive oral sex among homosexual and heterosexual males, the finding that prevalent HHV-8 infection tended to be associated with gonorrhea in both groups may indicate that this STI could be a biological marker of exposure to saliva during sex in both groups. The lack of statistically significant results may have been due to the limited power of the study resulting from the relatively small numbers of events.

The finding that the overall HHV-8 seroincidence (3.2/100 p-y) was more than three times higher than the overall HIV-1 seroincidence and that it was much higher than the seroincidence of HIV-1 and other STIs among both MSM and multiple-partner heterosexuals suggests that the circulation of HHV-8 may be sustained by practices other than those associated with the transmission of HIV-1 or other STIs.

Before drawing conclusions, some limits and biases of the study should be mentioned. First, not all the 72 individuals HHV-8 positive at enrollment were retested to confirm antibodies persistence over time. Nevertheless, the high proportion of confirmed positive results (98.1%) suggests that incidence rates were not biased by eventual seroreversion. Second, information on certain sexual practices involving contact with saliva, such as the frequency of oral-penile contact or oral-anal contact was no collected because not included in the individual behavioral data form aimed to HIV risk assessment. Third, the seroconversion rates may have been somewhat biased, given that persons who undergo repeated HIV-1 testing represent a self-selected population and to some extent may differ from those who do not return after the first test. Reasonably, we supposed that, in this study, the incidence rates of HHV-8 infection represent an estimate of infection among Mediterranean individuals who underwent repeated HIV-1 testing because of their sexual risk behavior.

Lastly, the multiple partner heterosexuals in this cohort may not have been representative of the general population of sexually active heterosexuals; thus the HHV-8 seroconversion rates may have been overestimated and the role of sexual transmission of infection inflated, especially for those with more once partner.

In conclusion, in our study population, the seroprevalence of HHV-8 infection was lower among multiple-partner heterosexuals than among MSM. The incidence of HHV-8 infection did not differ between MSM and multiple-partner heterosexual males, whereas a gender difference was found among heterosexuals. Among multiple-partner heterosexuals, the findings that HHV-8 infection tended to be associated with other STIs in the cross-sectional study and with an increased number of partners in the sero-incidence study seem to suggest that sexual behavior plays a role in transmission. Nonetheless, additional longitudinal studies will need to be conducted to better define the role of specific sexual practices in the increase of risk of HHV-8 infection.

## Competing interests

The author(s) declare that they have no competing interests.

## Authors' contributions

MG and GR conceived the study, supervised the data analysis and drafted the manuscript; PCF and CC carried out all the laboratory and serological assays; ADC and GP enrolled the study participants; SB contributed to performing the statistical analysis. All authors read and approved the final manuscript.

## Pre-publication history

The pre-publication history for this paper can be accessed here:


